# Iron acquisition and oxidative stress response in aspergillus fumigatus

**DOI:** 10.1186/s12918-015-0163-1

**Published:** 2015-04-24

**Authors:** Madison Brandon, Brad Howard, Christopher Lawrence, Reinhard Laubenbacher

**Affiliations:** Center for Cell Analysis and Modeling, University of Connecticut Health Center, 400 Farmington Ave, Farmington, 06030 USA; Center for Quantitative Medicine, University of Connecticut Health Center, Farmington, 06030 USA; Department of Biological Sciences, Virginia Tech, 1405 Perry Street, Blacksburg, 24061 USA; Virginia Bioinformatics Institute, Virginia Tech, 1015 Life Science Circle, Blacksburg, 24061 US; The Jackson Laboratory for Genomic Medicine, 10 Discovery Drive, Farmington, 06030 USA; Department of Cell Biology, University of Connecticut Health Center, 263 Farmington Ave, Farmington, 06030 USA

**Keywords:** Boolean network, Discrete dynamic model, Invasive aspergillosis, Siderophores, Stochastic discrete dynamical system

## Abstract

**Background:**

*Aspergillus fumigatus* is a ubiquitous airborne fungal pathogen that presents a life-threatening health risk to individuals with weakened immune systems. *A. fumigatus* pathogenicity depends on its ability to acquire iron from the host and to resist host-generated oxidative stress. Gaining a deeper understanding of the molecular mechanisms governing *A. fumigatus* iron acquisition and oxidative stress response may ultimately help to improve the diagnosis and treatment of invasive aspergillus infections.

**Results:**

This study follows a systems biology approach to investigate how adaptive behaviors emerge from molecular interactions underlying *A. fumigatus* iron regulation and oxidative stress response. We construct a Boolean network model from known interactions and simulate how changes in environmental iron and superoxide levels affect network dynamics. We propose rules for linking long term model behavior to qualitative estimates of cell growth and cell death. These rules are used to predict phenotypes of gene deletion strains. The model is validated on the basis of its ability to reproduce literature data not used in model generation.

**Conclusions:**

The model reproduces gene expression patterns in experimental time course data when *A. fumigatus* is switched from a low iron to a high iron environment. In addition, the model is able to accurately represent the phenotypes of many knockout strains under varying iron and superoxide conditions. Model simulations support the hypothesis that intracellular iron regulates *A. fumigatus* transcription factors, SreA and HapX, by a post-translational, rather than transcriptional, mechanism. Finally, the model predicts that blocking siderophore-mediated iron uptake reduces resistance to oxidative stress. This indicates that combined targeting of siderophore-mediated iron uptake and the oxidative stress response network may act synergistically to increase fungal cell killing.

**Electronic supplementary material:**

The online version of this article (doi:10.1186/s12918-015-0163-1) contains supplementary material, which is available to authorized users.

## Background

*Aspergillus fumigatus* is a ubiquitous airborne fungus which has become an increasingly dangerous pathogen of humans worldwide, causing invasive infections, severe asthma and sinusitis [1]. The most severe form of *A. fumigatus* infection, called invasive aspergillosis (IA), occurs when inhaled *A. fumigatus* spores germinate into hyphae and invade lung tissue. IA is a major cause of mortality in immunocompromised human hosts [2-6]. In immunocompetent individuals *A. fumigatus* may trigger allergic reactions and is a major cause of fungal keratitis, an inflammation of the cornea [7].

Our focus on *A. fumigatus* oxidative stress response and iron acquisition is motivated by the following three arguments. First, several studies show that deletion of genes involved in either *A. fumigatus* oxidative stress response or iron acquisition leads to attenuated virulence *in vivo* [5,8-10]. Impairment of the corresponding host defense mechanisms, e.g. defective ROS production or inability to sufficiently deplete available iron, also leads to an increased susceptibility to *A. fumigatus* infection [4,10,11]. Second, recent publications present proof of concept that targeting either *A. fumigatus* oxidative stress response or iron acquisition systems may be an effective treatment strategy [10,12]. Thus oxidative stress response and iron acquisition are important systems contributing to *A. fumigatus* pathogenicity, and both systems are feasible targets for therapeutic intervention. Third, iron uptake and oxidative stress response networks are known to interact, and hence more can be learned about the molecular mechanisms underlying these networks if they are studied together. In fact, a connection between iron uptake and oxidative stress response has been described in both *A. fumigatus* and *S. cerevisiae* [13-15]. These motivating points will now be discussed in greater detail.

Several lines of evidence point to the *A. fumigatus* ROS-detoxifying enzymes as key virulence factors and potential drug targets. Firstly, on the host side, the activation of the enzymatic complex NADPH oxidase (NOX) and subsequent production of cytotoxic ROS by host phagocytic cells is a critical mechanism for host defense against fungal pathogens such as *A. fumigatus* [16-18]. Noteably, a mouse model of fungal keratitis in the cornea using mice that do not express a functional NOX complex showed that neutrophil NOX expression was required for inhibiting *A. fumigatus* growth [10]. From a fungal perspective, genes encoding oxidative stress response enzymes are known to be among the most differentially expressed genes of *A. fumigatus* hyphae following exposure to human neutrophils from healthy individuals [19]. Furthermore, *A. fumigatus* antioxidant enzymes and the ROS-sensing transcription factor deletion strains show a heightened sensitivity to ROS *in vitro* [9,10,20].

Other evidence suggests that the adeptness of *A. fumigatus* to acquire iron from the host is a major basis of its pathogenicity. Both the fungus and host require iron for important cellular functions including respiration, gene regulation, DNA synthesis, and oxidative stress response [21]. Iron deprivation of invading pathogens by the host is a crucial host defense mechanism [22-24]. To combat this, fungi secrete siderophores, low-molecular-mass iron binding compounds that sequester iron from host proteins. [25]. A significant body of evidence suggests that the victor of this battle for iron is a key determinant of whether infection will persist or be cleared [25-28]. Notably, a mutant *A. fumigatus* strain unable to produce both extra- and intracellular siderophores was avirulent in a mouse model of IA [5]. Any advantage *A. fumigatus* has in the battle for iron can be dangerous. For instance, increased iron in bone marrow is a risk factor for IA in high-risk patients [11]. Similarly, the heightened susceptibility to fungal infections in neutropenic patients may be in part due to increased extracellular iron due to the absence of host cells which mediate iron sequestration [29].

Leal *et al*. show that the use of topical drugs to target either *A. fumigatus* oxidative stress response or iron acquisition systems is effective for treating *A. fumigatus* infection in mice cornea [10,12]. The fungal iron acquisition system is a particularly promising target for therapeutic intervention because the fungal proteins which import ferri-siderophores are one of the few protein families that are unique to fungi [30]. This might make it possible to design drugs which specifically target the fungus without affecting the host, perhaps by a “Trojan horse” approach [31,32]. Furthermore, the iron acquisition and oxidative stress response networks are connected. Indeed it was found in *A. fumigatus* that deletion of a key iron regulatory protein, *ΔsreA*, caused increased sensitivity to superoxide [13]. Also in *A. fumigatus* deletion of an intracellular siderophore led to decreased expression of conidial, but not hyphal, catalase [33]. Similarly, in *A. nidulans* oxidative stress was shown to increase the accumulation of an intracellular siderophore [14]. Finally, a yeast mutant with deletion of genes that regulate the transcription of high-affinity iron transport genes also showed several phenotypes related to oxidative stress such as hypersensitivity to hydrogen peroxide [15].

### The role of mathematical modeling

The purpose of the present work is to gain a deeper understanding of the molecular mechanisms underlying the systems that most contribute to *A. fumigatus* pathogenicity, the iron acquisition and oxidative stress response networks. For this purpose, we have constructed a novel dynamic mathematical model of key molecular interactions defining these networks. Mathematical modeling of complex molecular interaction networks allows for the encoding of dynamic interactions among molecules, and thus enables the simulation of global network behavior based on information known about individual interactions.

Recently, the first computational model of *A. fumigatus* iron regulation was proposed [34]. Taking a top-down approach, Linde et al. used gene expression time series data to reverse engineer a regulatory network and predict new interactions between transcription factors and target genes. The authors constructed a system of differential equations to model changes in gene expression as a function of other genes in the network. A major challenge to building differential equations models is that many of the required parameters are either unknown or unmeasurable, and so parameters must be estimated by fitting equations to experimental time series data, which is limited for *A. fumigatus* iron regulation and oxidative stress response.

However, there is a wealth of qualitative data for these networks, for example the interaction between a transcription factor and a gene, from high-throughput transcriptomic experiments such as microarrays [13,29,35]. In contrast to the Linde et al. computational study, we take a bottom-up approach to investigate both iron regulation and oxidative stress response, and we apply a discrete dynamic modeling framework. Discrete models make use of the available qualitative data by encapsulating the regulatory logic driving a network, and they do not require kinetic parameters. Simulation of discrete models provides coarse-grained information as the network evolves over an arbitrary unit of time in response to broad changes in some physiological condition. Qualitative observations generated by these models are extremely useful for investigating the ability of known or proposed information to explain current experimental results, studying how perturbations may alter global behavior, and for pinpointing productive future experiments.

Discrete models, in particular Boolean network models, are routinely used to investigate biological systems such as gene regulatory networks, signaling pathways, and metabolic pathways [36-41]. Discrete models have contributed insights into host-pathogen interactions for several pathogenic bacteria [42-44]. To our knowledge, discrete models have not yet been used to study *A. fumigatus* biology, yet many aspects of yeast biology have been explored via discrete models [45-47]. This includes a Boolean network model of metabolic adaptation to oxygen in relation to iron homeostasis and oxidative stress [48].

## Results and discussion

### Description of model species

**Figure 1:**
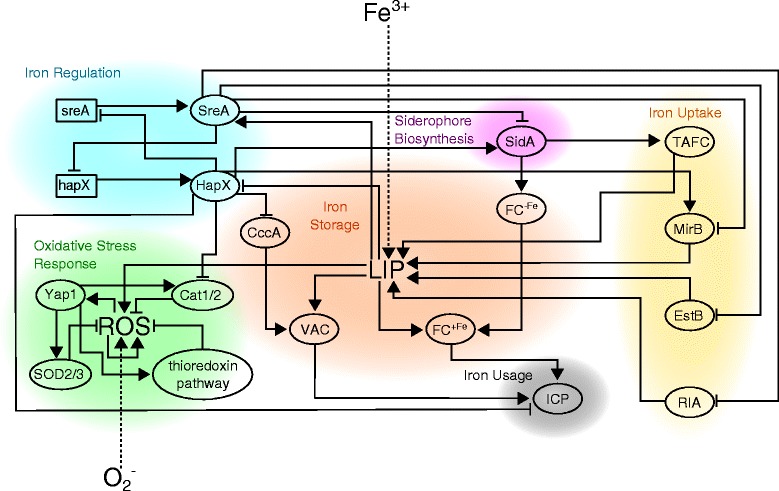
Model interaction diagram of *A. fumigatus* iron regulation and oxidative stress response. Rectangles represent genes. Ovals represent other molecules. Fe ^3+^ and O$_{2}^{-}$ are external parameters to describe the physiological state of a fungal cell. *A*→*B* represents activation. *A*⊣*B* represents inhibition.

The model contains an oxidative stress response module and a larger iron acquisition module which is made up of five submodules: siderophore biosynthesis (SB), iron uptake, iron storage, iron usage, and iron regulation. Figure 1 is a graphical representation of all model species (nodes), their interactions (edges), and the sign of the interaction.

#### Siderophore biosynthesis

*A. fumigatus* produces four siderophores, low molecular mass ferric iron-specific chelators [33]. Two extracellular siderophores are excreted from the cell to sequester iron from the extracellular space [8]. And two intracellular siderophores are used for intracellular iron storage [14,49]. For simplicity, our model considers only one extracellular siderophore, triacetylfusarinine C (TAFC), and one intracellular siderophore, ferricrocin (FC), which have been shown to be the two most abundant and active *A. fumigatus* siderophores [50]. The first step in the biosynthesis of all four siderophores is the hydroxylation of ornithine catalyzed by SidA, an ornithine monooxygenase.

#### Iron uptake

Iron uptake is believed to be the main iron homeostasis control mechanism used by *A. fumigatus*, in part because mechanisms of iron excretion have not been found in fungi [51]. *A. fumigatus* has three known mechanisms of iron uptake: low affinity ferrous iron uptake, which has not yet been characterized at the molecular level, and two high affinity ferric iron uptake systems, namely siderophore-mediated iron uptake and reductive iron assimilation (RIA) [8]. RIA involves the reduction of ferric iron to ferrous iron by the ferric reductase FreB and subsequently the import of ferrous iron by a protein complex consisting of the ferroxidase FetC and the iron permease FtrA [52]. For simplicity, these three proteins are modeled as a single species called RIA.

Siderophore-mediated iron uptake is represented in the model by nodes TAFC, MirB, and EstB. TAFC is released into the extracellular space to steal ferric iron from host proteins such as transferrin [53]. A protein family called siderophore-iron transporters (SIT) recognizes and retrieves specific ferri-siderophores. After binding to Fe ^3+^, the ferri-TAFC complex is taken back up by the TAFC-specific SIT MirB [54]. After import into the cell the ferri-TAFC complex is degraded by a TAFC-specific esterase called EstB [55]. Subsequently, breakdown products are recycled, and iron is released into the cell for transfer to intracellular siderophores or the iron vacuole [56].

#### Iron storage

Unlike bacteria, plants and animals, most fungi lack ferritin-mediated iron storage [51]. Instead, *A. fumigatus* relies on siderophore-mediated iron storage via the intracellular siderophore FC and a siderophore-independent iron storage unit, the iron vacuole [49,56]. Import of iron into the vacuole is in part mediated by the protein CccA which is localized in the vacuolar membrane [56]. The labile iron pool, a pool of redox-active iron, is also modeled as a transitory state between the release of iron from ferri-TAFC and the transfer of iron to FC or the vacuole. Again, since fungi lack mechanisms for iron excretion, iron storage plays a crucial role in avoiding iron-induced toxicity. In *A. nidulans*, FC deficiency was shown to cause an increase in LIP and a decrease in the oxidative stress resistance of hyphae [57].

#### Iron usage

All iron consuming pathways, for example heme biosynthesis, TCA cycle, respiration, and ribosome biogenesis, are modeled as a single species named ICP.

#### Regulation

Iron is toxic in excess; thus tight regulatory mechanisms are required to maintain iron homeostasis. Iron regulation in *A. fumigatus* is controlled by two central transcription factors: the bZip CCAAT-binding transcription factor HapX and the GATA transcription factor SreA [13,29]. HapX and SreA are postulated to sense intracellular iron levels through a posttranslational mechanism similar to the mechanism employed by a closely related species, the fission yeast *Schizosaccharomyces pombe* [27]. In *S. pombe*, orthologs of HapX and SreA physically interact with a monothiol glutaredoxin Grx4 which is localized along the nuclear rim [58-60]. When intracellular iron levels are low Grx4 maintains SreA in an inactive state [59]. When intracellular iron levels are high, Grx4 inactivates HapX by directing its export from the nucleus [58]. Hence, intracellular iron blocks HapX function while activating SreA function at the posttranslational level. Furthermore, SreA represses transcription of *hapX* when intracellular iron levels are high, while HapX represses transcription of *sreA* when intracellular iron levels are low. Both transcriptional and posttranslational regulatory mechanisms are modeled.

SreA transcriptionally represses genes coding for proteins involved in iron uptake, including *sidA*, *mirB*, *estB*, and those involved in RIA [13]. HapX activates siderophore biosynthesis, in part by upregulating the production of the precursor ornithine, and activates the transcription of *mirB* [29]. HapX indirectly activates the transcription of *sidA*, *estB*, and the genes involved in RIA through its repression of *sreA*. Additionally, HapX represses iron consuming pathways, *cat1*, and *cccA* at the transcriptional level.

#### Oxidative stress response

NOX expressed by host phagocytic cells catalyzes the conversion of oxygen to the the extremely reactive superoxide anion, O$_{2}^{-}$. Contact between neutrophils and hyphae triggers a respiratory burst, the targeted release of O$_{2}^{-}$ from the neutrophil into the extracellular space where it diffuses into nearby hyphal cells. The *A. fumigatus* ROS-sensing transcription factor Yap1 is believed to be the main regulator of antioxidant defense against O$_{2}^{-}$ and hydrogen peroxide, H _2_0_2_ [61,62]. Yap1 typically resides in the cytoplasm, yet under oxidative stress conditions Yap1 localizes to the nucleus and from there controls, directly or indirectly, the expression of key ROS-detoxifying enzymes including superoxide dismutases (SODs), catalases, and thioredoxin peroxidases (peroxiredoxins) [61]. Elevated free iron levels (high LIP) in the cell also contribute to the formation of ROS [63].

SODs catalyze the conversion of O$_{2}^{-}$ to less reactive H _2_*O*_2_ which can then be converted to non-reactive H _2_O by either catalases or peroxiredoxins. *A. fumigatus* produces four SODs, yet only the mitochondrial SOD2 and cytoplasmic SOD3 are modeled here since both are most strongly expressed in hyphae, the tissue invasive form of this pathogen, as opposed to in conidia [20]. *A. fumigatus* hyphae produce two catalases, Cat1 and Cat2, which break down hydrogen peroxide [9]. The thioredoxin pathway in *A. fumigatus* is not well characterized; however, two putative peroxiredoxins and five putative thioredoxins have been identified [10,61]. Briefly, peroxiredoxins reduce H _2_0_2_ and by doing so become oxidized, a non-functional state. Thioredoxins then reduce the oxidized peroxiredoxins back to their functional state so that more H _2_0_2_ can be reduced [64]. In the model the thioredoxin pathway is modeled as a single variable. Note that in Figure 1 the ROS species has a self-activating arrow. The purpose of this interaction is to enforce “memory” in the system, i.e. if ROS is high at the current time step and antioxidant enzymes are not expressed or inactive, then the ROS variable should “remember” to remain high until antioxidant enzymes are active.

### Building and simulating the mathematical model

The model presented in this paper is discrete. This means species can take on only a finite number of states, and the state of each species is iteratively updated at discrete time steps according to logical rules. The discrete model presented here is a Boolean network model, meaning that each species can take on only two states (e.g. low expressed or high expressed; low active or high active), which may be represented numerically by either a 0 or a 1. Furthermore, the rules determining how species are updated are Boolean functions.

We conducted an extensive literature survey to identify key species involved in the *A. fumigatus* iron regulatory and oxidative stress response networks as well as the interactions of each species with other species in the networks (Figure 1). Table 1 gives a biological description of each species and the meaning assigned to its states. Note that for different species we may assign different meanings to their states. Importantly, the two species iron and superoxide should be thought of as external parameters since they are meant to distinguish between different physiological conditions that are reflective of the host-pathogen interaction. Iron and superoxide have no regulators (incoming arrows) (see Figure 1) and so, unlike other species, a fixed state is chosen at the start of a simulation and this state will never be updated.

**Table 1:** **List of species, their biological type, and their model states**

**Species**	**Type**	**Model states**	
		**0**	**1**
*hapX*	Gene	Low expressed	High expressed
*sreA*	Gene	Low expressed	High expressed
HapX	Protein; bZip CCAAT-binding TF	Low active	High active
SreA	Protein; GATA TF	Low active	High active
RIA	Enzyme complex; reductive iron assimilation	Low active	High active
EstB	Enzyme; TAFC-specific esterase	Low active	High active
MirB	Protein; TAFC-specific importer	Low active	High active
SidA	Enzyme; ornithine monooxygenase	Low active	High active
TAFC	Extracellular siderophore	Low synthesized	High synthesized
ICP	Iron consuming pathways	Low active	high active
LIP	Labile iron pool	Low iron	High iron
CccA	Protein; iron importer to vacuole	Low active	High active
FC ^+*Fe*^	Intracellular siderophore w/ bound iron	Low iron	High iron
FC ^−*Fe*^	Intracellular siderophore w/o bound iron	Low synthesized	High synthesized
VAC	Vacuole	Low iron	High iron
ROS	Reactive oxygen species	Low ROS	High ROS
Yap1	Protein; bZip TF	Low active	High active
SOD2/3	Enzyme; superoxide dismutase	Low active	High active
Cat1/2	Enzymes; hyphal catalases	Low active	High active
Thioredoxin P.	Enzyme pathway	Low active	High active
Iron	Physiological state	Low iron	High iron
Superoxide	Physiological state	Low superoxide	High superoxide

Next we integrated all identified interactions into a dynamic framework by specifying, through logical rules called update rules, how each species transitions between its two states based upon the states of its inputs. Table 2 lists the update rule for each species as a Boolean function along with a summary of experimental support for each rule. The model is available in SMBL qual format, a standard language for representation of qualitative models of biological networks [65], see Additional file 1.

**Table 2:** **Update rules of model species and supporting literature citations**

**Update rules**	**Literature support**
1 *hapX*(t+1) = NOT SreA	Transcription of *hapX* is repressed by SreA [13,29].
2 *sreA*(t+1) = NOT HapX	Transcription of *sreA* is repressed by HapX [13,29].
3 HapX(t+1) =*hapX* AND (NOT LIP)	An ortholog of HapX is inactivated by intracellular iron [58].
4 SreA(t+1) =*sreA* AND LIP	An ortholog of SreA is activated by intracellular iron [59,60].
5 RIA(t+1) = NOT SreA	SreA transcriptionally represses RIA genes [13].
6 EstB(t+1) = NOT SreA	SreA transcriptionally represses *estB* [13].
7 MirB(t+1) = HapX AND (NOT SreA)	HapX transcriptionally activates *mirB* [29]. SreA transcriptionally represses *mirB* [13].
8 SidA(t+1) = HapX AND (NOT SreA)	HapX up regulates the SidA substrate ornithine [29]. SreA transcriptionally represses *sidA* [13].
9 TAFC(t+1) = SidA	SidA catalyzes the first step in siderophore biosynthesis [5,8]
10 ICP(t+1) = (NOT HapX) AND (VAC OR FC ^+*Fe*^)	HapX represses consumption of intracellular iron [29].
11 LIP(t+1) = (TAFC AND MirB AND EstB) OR (Iron AND RIA)	TAFC sequesters iron from the extracellular space [8]. MirB imports ferri-TAFC [54]. EstB
	degrades ferri-TAFC bonds and releases free iron [55]. RIA compensates for a lack of
	siderophores when grown in high iron media [33].
12 CccA(t+1) = NOT HapX	HapX transcriptionally represses *cccA* [29].
13 FC ^−*Fe*^(t+1) = SidA	SidA catalyzes the first step in siderophore biosynthesis [5,8]
14 FC ^+*Fe*^(t+1) = LIP AND FC ^−*Fe*^	FC is involved in intracellular iron storage [14,49].
15 VAC(t+1) = LIP AND CccA	CccA mediates import of intracellular iron into the vacuole [56].
16 ROS(t+1) = LIP OR	Elevated free iron levels catalyze the formation of ROS [63].
$\left ({\vphantom {\sum \limits ^{1}_{1}}}\; \text {Superoxide AND}\; \left ({\vphantom {\frac {\sum }{\sum }}}\; \text {NOT (SOD3 AND ThP AND Cat1/2)}\; \right)\; \right)$ OR	SODs convert O$_{2}^{-}$ to H _2_ *O*_2_ [20]. Either catalases or thioredoxin
$\left ({\vphantom {\sum \limits ^{1}_{1}}}\; \text {ROS AND} \left ({\vphantom {\frac {\sum }{\sum }}}\; \text {NOT} \left ({\vphantom {\frac {1}{2}}}\; \text {SOD3 AND (ThP OR Cat1/2)}\; \right)\; \right)\; \right)$	convert H _2_ *O*_2_ to non-reactive *H*_2_O [9,64].
17 Yap1(t+1) = ROS	Yap-1 is activated by superoxide [61,62].
18 SOD2/3(t+1) = Yap1	Yap-1 activates transcription of *sod2/3* [61].
19 Cat1/2(t+1) = Yap1 AND (NOT HapX)	Yap-1 activates transcription of *cat1/2* [61]. HapX transcriptionally represses *cat1* [29].
20 ThP(t+1) = Yap1	Yap-1 activates transcription of thioredoxin peroxidases [61].
21 Iron(t+1) = Iron	External parameter.
22 Superoxide(t+1) = Superoxide [ = NOT Superoxide, Figure 5 only]	External parameter.

Species that appear on the right side of the = represent states at time t.

**Figure 2:**
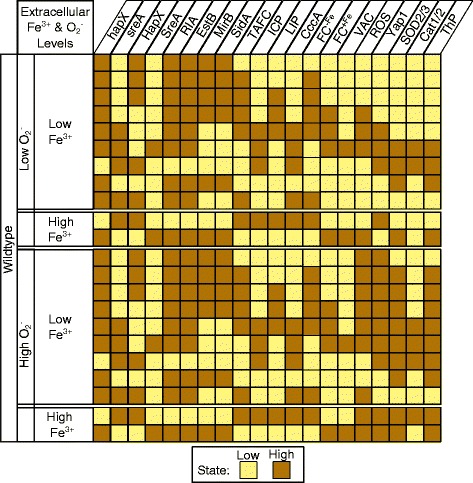
Stable states of *A. fumigatus* iron regulatory and oxidative stress response networks. This figure shows the cyclic attractor for each of the four possible external conditions. States transition from top to bottom. Under both low iron conditions ICP is in state 0 (low) the majority of the cycle. Under both high superoxide conditions ROS is in state 1 (high) the majority of the cycle.

In general, the dynamic behavior of discrete models is simulated by starting from an initial state and then enumerating the changing state space as each species is updated over a specified number of iterations called time steps. The result of deterministic simulations, when all species are updated simultaneously at each time step, is shown in Figure 2. This system has no steady state solution for any of the four external conditions. All long term behavior is oscillatory, i.e. the stable states form a limit cycle and 100 *%* of the 1048576 states converge to the limit cycle displayed.

Many biological processes such as gene expression have been found to exhibit a high degree of stochasticity [66-69]. Furthermore, protein levels can differ significantly among cells in a population [70,71]. To our knowledge no single cell gene expression or protein level measurements are available for *A. fumigatus*. Hence in order to make comparisons to experimental data possible, we needed to account for the variability that one observes in a population of cells. We accounted for this variability by simulating randomness in the update of species. At each time step, rather than updating all species, some species are randomly selected to be updated, while the unselected species are left in their current state. We assume that the average of many of these stochastic simulations represents a population level measurement.

### Linking model simulation results to phenotype predictions

**Figure 3:**
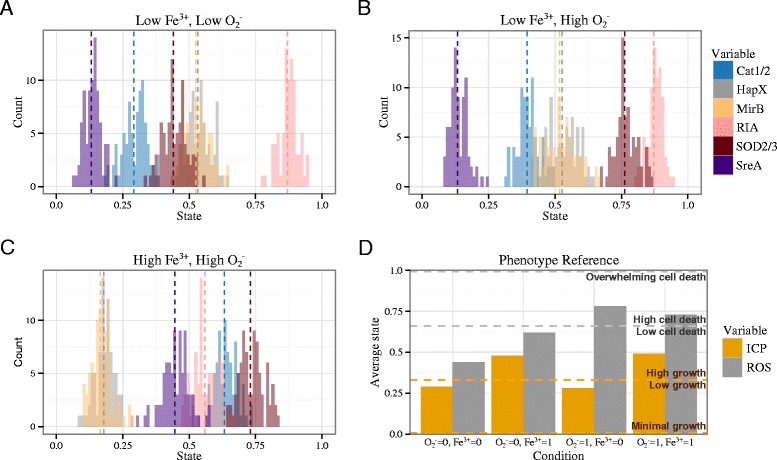
Summary of model wild type phenotypes.**(A)-(C)** Histogram of average states of six species from time steps 100-200 (i.e., the model reaches a stable configuration before counting begins). Vertical dashed lines mark stable distribution means (SDM). **(D)** The SDM of ICP and ROS for a wild type fungal cell under each of the four conditions overlayed with a depiction of the phenotype reference. If the SDM of ICP is 0, then we interpret the model observation as minimal or no growth. An ICP SDM in (0, 0.33) is interpreted as low growth. Otherwise, an ICP in [0.33, 1] signifies a high growth phenotype. If the ROS SDM falls in [0.66,1] we interpret this as high cell death. When the SDM of ROS is 1, we assume the ROS is so overwhelming that the entire population dies. Otherwise, for an ROS SDM in [0, 0.66) the interpretation is low cell death.

For the results presented in this paper, we ran 100 independent stochastic simulations (initialized in the same state) and, for each species, calculated the average state at each time step. From there, we counted the number of times a species took on any average state throughout the simulation period. We can plot a histogram of these counts to visualize a distribution of species’ average state across 100 simulations, as in Figure 3. To characterize long-term behavior, we introduce a measure called the stable distribution mean (SDM) for a given species under a given set of initial conditions. The SDM is simply the mean of the distribution of the average states from time steps 100 to 200. Excluding the first 100 time steps from the calculation gives the model time to settle into a stable configuration.

**Figure 4:**
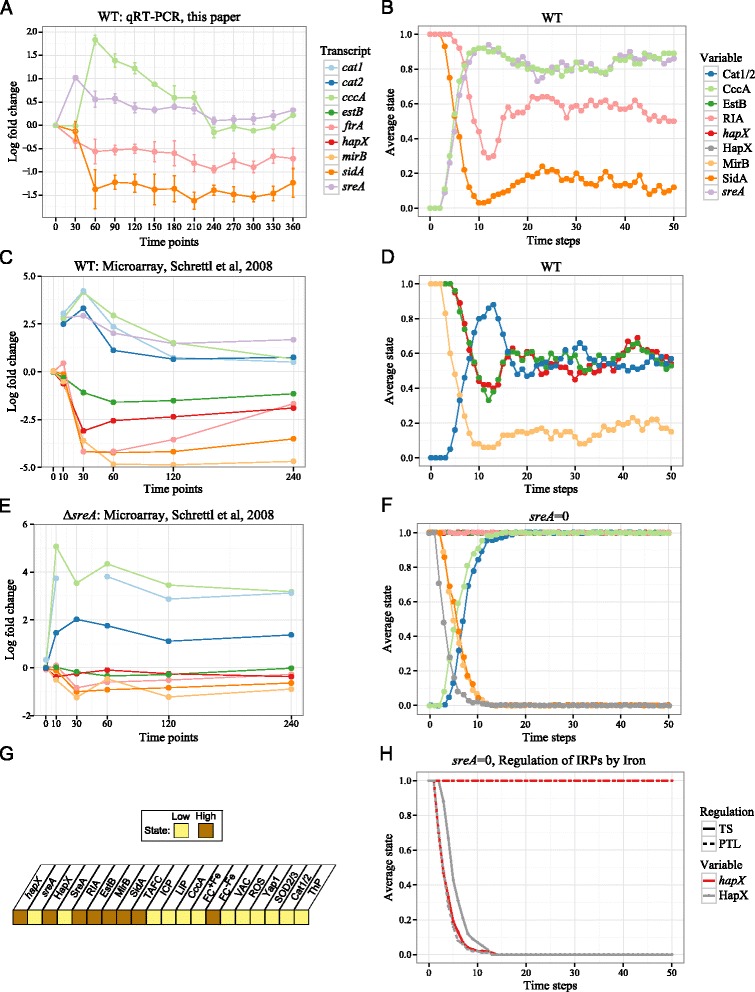
Model simulation results and experimental time course data following a switch from low iron, low superoxide to high iron, low superoxide conditions.**(A)** Gene expression from a qRT-PCR experiment conducted in this study. **(C), (E)** Gene expression from a microarray experiment by Schrettl et al., 2008 for a wild type and *Δ* *sreA* strain, respectively [13]. **(B), (D), (F), (H)** Simulated trajectories for corresponding model species plotted as the average state at each time step across 100 stochastic simulations. **(G)** All simulations were initialized from this state representing iron starvation. In **(H)** trajectories are generated by a model with post-translational regulation of HapX and SreA by iron (PTL) and an altered model with transcriptional regulation of *hapX* and *sreA* by iron (TS).

We first simulated the Boolean network model of wild type *A. fumigatus* under each of the four possible conditions: (1) low iron and low superoxide, (2) high iron and low superoxide, (3) low iron and high superoxide, and (4) high iron and high superoxide. Figure 3 (A) - (C) show the distributions of average states across 100 wild type simulations for six selected species under three of the four conditions. Wild type distributions are not shown for the remaining condition; instead Figure 4(B) and (D) show trajectories, the average state at each time step, for eight selected species.

For both low iron conditions, we observed that HapX, the transcription factor activating iron uptake and repressing iron consumption, is more active than SreA, the transcription factor repressing iron acquisition. This leads to strong activity of proteins related to siderophore-mediated iron uptake (MirB) and reductive iron assimilation (RIA). Conversely, for the high iron conditions we observe that SreA is more active than HapX. Consequently, activity of both MirB and RIA are significantly reduced as compared to the low iron, low superoxide condition. These results recapitulate experimental observations [29,72].

ROS-detoxifying enzymes, SOD2/3 and Cat1/2, are moderately active in the low iron, low superoxide condition. As expected, since free iron and superoxide contribute to ROS, the activity of SOD2/3 and Cat1/2 are elevated in high iron and high superoxide conditions. Further, we observed that in low iron conditions Cat1/2 is less active than SOD2/3. This makes sense because catalases require heme as a cofactor whereas SODs instead require copper, zinc or manganese [20].

Based on experimental results of wild type *A. fumigatus* growth under each of the four conditions [8,20,33], we used the stable distribution mean (SDM) of model variables ROS and ICP to establish a phenotype reference according to the following rules (see Figure 3). If the SDM of ICP is 0, then we interpret the model observation as minimal or no growth. An ICP SDM in (0, 0.33) is interpreted as low growth. Otherwise, an ICP in [0.33, 1] signifies a high growth phenotype. If the ROS SDM falls in [0.66,1] we interpret this as high cell death. When the SDM of ROS is 1, we assume ROS is so overwhelming that the entire population dies. Otherwise, for an ROS SDM in [0, 0.66) the interpretation is low cell death. These rules bin wild type model behavior to match what we observe experimentally. We then used this set of rules to infer the severity of model knockouts.

### Stochastic simulations reproduce *in vitro* time course data

We validated the model on the basis of its ability to reproduce transcriptional time course data from a previously published study and data generated in this study. In both experiments, *A. fumigatus* is grown in iron depleted minimal media (low iron, low superoxide conditions). After an incubation period, iron is added to the media (high iron, low superoxide conditions) and gene expression is measured over a period of hours either using microarrays (Schrettl et al., 2008 [13]) or by qRT-PCR (this study, see Methods).

To mimic the switch from low iron to high iron conditions, all simulations were initialized from a state of iron starvation (Figure 4G). Iron and superoxide were fixed at 1 and 0, respectively, throughout model simulations. Experimental results are displayed alongside model simulation results in Figure 4. From the Schrettl et al. study, we plot all time course data for genes which correspond to species in the model. For knockout simulations, the state of the corresponding species is fixed at 0. To distinguish between model and experimental knockouts, using *sreA* as an example, we write *sreA*=0 to refer to model knockouts and *ΔsreA* to refer to experimental knockouts.

The model provides a good qualitative reproduction of changes in gene expression over time. Additionally the model captures the relative differences in degrees of up- or down-regulation among genes. For ease of exposition, we discuss the following results in the syntax of model species even though some model species refer to amount of protein while the experimental results refer to amount of transcript.

#### Wild type results

For wild type *A. fumigatus*, the experimental and model simulation results show the same expression patterns. Following the switch from low to high iron, catalases Cat1/2, the vacuolar iron importer CccA, and *sreA* were quickly up-regulated, then slowly decreased and leveled off. After the addition of iron, the expression of *hapX*, siderophore biosynthesis enzyme SidA, ferri-siderophore importer MirB, ferri-siderophore esterase EstB, and reductive iron assimilation RIA (*ftrA* in the experimental data) were quickly down-regulated and remained low. Moreover, the model recapitulates experimental observations that among species contributing to iron uptake, SidA and MirB were less active under high iron conditions, as compared to the activity of RIA and EstB.

#### *ΔsreA* results

Experimental and model simulation results are also in agreement for the *ΔsreA* deletion strain, except for SidA and MirB which we discuss shortly. As in the wild type case, expression of CccA and Cat1/2 increased sharply after the addition of iron. However in the *ΔsreA* knockout, expression of CccA and Cat1/2 remained high over time. This can be attributed to the fact that iron uptake mechanisms were depressed in a *ΔsreA* mutant. Indeed, in contrast to the wild type case, both experimental and model simulation results showed no change in the expression of *hapX*, EstB, or RIA despite being exposed to high iron over a long period of time.

In the *ΔsreA* deletion experimental data the amount of *sidA* transcript remained the same, whereas the amount of SidA enzymatic activity in the model simulation plummeted to very low. This in fact is not a discrepancy and serves to illustrate an important point. Although both HapX and SreA ultimately activate and respectively repress SidA enzymatic activity, only SreA directly transcriptionally regulates *sidA* [13,29]. Instead, HapX up-regulates the production of ornithine, the SidA substrate. This explains the derepression of *sidA* in the experimental data yet the lack of SidA activity in the model simulation. A difference between amount of *sidA* transcript and SidA enzymatic activity is not visible in the wild type data because HapX indirectly regulates the transcription of *sidA* through repression of *sreA*; this feature is lost in a *ΔsreA* mutant.

The discrepancy between gene expression of *mirB* in the experiment and activity of MirB in the model is unexpected since MirB is known to be regulated transcriptionally by both HapX and SreA [13,29]. This may suggest that MirB is in fact not regulated transcriptionally by HapX. Or alternatively, since MirB is known to transport other siderophores it may have additional regulators [54].

#### Regulation of HapX and SreA by iron

The post-translational regulation of *S. pombe* orthologs of HapX and SreA by iron has been investigated [58-60]. *A. fumigatus* HapX and SreA are postulated to sense intracellular iron levels through a similar post-translational mechanism, but the corresponding mechanism has not yet been identified [27]. We included in the model both the known transcriptional regulation of *hapX* and *sreA*, by SreA and HapX respectively, and the proposed but not yet verified post-translational regulation of HapX and SreA by intracellular iron. Additionally, we analyzed a modified model whereby *hapX* and *sreA* were regulated transcriptionally by iron, and all other interactions are the same. Both versions of the model were consistent with gene expression data for the wild type. However, the model with HapX and SreA regulated by iron at the post-transtlational level, but not the modified model, agreed with the Schrettl et al. *hapX* gene expression data for the *ΔsreA* mutant strain (see Figure 4H). This provides support for the hypothesis that, as in *S. pombe*, a post-translational regulation of the iron regulatory proteins HapX and SreA by iron is in fact employed by *A. fumigatus*.

### Model knockout simulations recapitulate experimental gene deletion results.

Next, we systematically analyzed the effect of all single and double knockouts on model predicted phenotypes under each of the four external conditions. Key observations from wild type and knockout simulations are summarized in Table 3.

**Table 3:** **Summary of observations from model wild type and knockout simulations**

**Condition**	**Strain**	**Long term model behavior**			**Cell**	**Cell**	**Support**	**Conflicts**
		**ICP**	**ROS**	**Other interesting behavior**	**Growth**	**Death**		
O$_{2}^{-}=0$	wt	0.29	0.44	∙ TAFC = *FC* ^−*Fe*^=0.52; high siderophore production	−	−	[5,8]	
				∙ Cat1/2 = 0.29; SOD2/3 = *ThP* = 0.44			prediction	
				∙ FC ^−*Fe*^/*FC* ^+*Fe*^=1.7			[14]	
Fe ^3+^=0	*hapX* = 0	0	0	∙ SidA = 0	−	−	[29]	
	SidA = 0 or TAFC = 0	0	0		−	−	[5,8,33,52]	
	FC ^−*Fe*^=0	0.11	0.43		−	−	[14,33]	
	EstB = 0 or MirB = 0	0	0	∙ TAFC = *FC* ^−*Fe*^ = 1; accumulation of siderophores	−	−	[55]	
	Yap1 = 0 or SOD2/3 = 0	0.29	1		−	+		[10,20]
O$_{2}^{-}=0$	wt	0.48	0.62	∙ TAFC = *FC* = 0.18; low siderophore production	+	−	[5,8]	
				∙ Cat1/2 = 0.54, SOD2/3 = *ThP* = 0.61			[13]	
				∙ FC ^−*Fe*^/*FC* ^+*Fe*^=1			[14]	
Fe ^3+^=1	*sreA* = 0	1	1	∙ Derepression of *hapX*, RIA, CccA, & Cat1/2	+	+	[13,52]	
				∙ LIP = *VAC* = 1; iron overload			[13]	
	SidA = 0	0.42	0.61		+	−	[5,8,33]	
	RIA = 0	0.29	0.45	∙ SidA = 0.52; increased siderophore production	−	−	[8,33]	
	SidA = *RIA* = 0	0	0		−	−	[8,33]	
	Yap1 = 0 or SOD2/3 = 0	0.49	1	∙ Decreased resistance to Fe ^3+^	+	+		[10,20]
O$_{2}^{-}=1$	wt	0.28	0.78	∙ SOD2/3 = *ThP* = 0.76; Cat1/2 = 0.40	−	+	[20,33,61]	
	*hapX* = 0	0	0.58	∙ Derepressed Cat1/2 and increased resistance to O$_{2}^{-}$	−	−	prediction	
Fe ^3+^=0	SidA = 0, TAFC = 0,	0	1	∙ Decreased resistance to O$_{2}^{-}$	−	+	prediction	
	MirB = 0 or EstB = 0							
	Yap1 = 0, SOD2/3 = 0,	0.29	1	∙ Decreased resistance to O$_{2}^{-}$	−	+	[9,10,20,61]	
	Cat1/2 = 0 or ThP = 0							
O$_{2}^{-}=1$	wt	0.49	0.73	∙ FC ^−*Fe*^/FC ^+*Fe*^=1	+	+	[14,20,33]	
				∙ SOD2/3 = *ThP* = 0.73; Cat1/2 = 0.63			prediction	
Fe ^3+^=1	*sreA* = 0	1	1	∙ Decreased resistance to Fe ^3+^	+	+	[13]	
	Yap1 = 0, SOD2/3 = 0,	0.47	1	∙ Decreased resistance to Fe ^3+^ and O$_{2}^{-}$	+	+	[9,20,61]	
	Cat1/2 = 0 or ThP = 0							

Numerical values in the ‘Long Term Model Behavior’ column represent SDMs. A − denotes low cell growth or low cell death, while a + denotes high cell growth or high cell death. Citations for supporting and conflicting literature are provided.

#### Iron regulation knockouts

For the low iron conditions, the SidA = 0 knockout led to minimal or no growth (SDM of ICP = 0). However, no growth defects were observed when RIA = 0 under the same conditions. In high iron conditions, the SidA = 0 knockout did not deviate from the wild type high growth phenotype. However, in high iron conditions, a double RIA = *SidA* = 0 knockout led to minimal or no growth. These results are consistent with experimental results showing: (1) a *ΔsidA* but not *ΔftrA* mutant is avirulent in a mouse model of aspergillosis [5,8], and (2) that RIA can compensate for a lack of siderophores in high iron but not low iron conditions [33].

Also in agreement with experiments, under low iron conditions the TAFC = 0 knockout led to more severe growth defects than the FC^−*Fe*^ = 0 knockout [33]. Knocking out any part of the siderophore iron uptake system under low iron conditions (TAFC =0, MirB =0, or EstB =0) resulted in a minimal growth phenotype. Interestingly, we observed an accumulation of siderophores for either MirB = 0 or EstB = 0 under low iron conditions, a behavior which has been observed experimentally in an *ΔestB* mutant [55].

The *hapX* = 0 knockout displayed a minimal growth phenotype under low iron conditions, but had no defects under high iron conditions. Conversely, the *sreA* = 0 knockout led to iron overload (SDM of LIP = 1) and cell death by overwhelming ROS (SDM of ROS = 1) under high iron conditions, but had no defects under low iron conditions. This recapitulates experimental results showing that growth defects of a *ΔhapX* mutant are confined to low iron conditions while growth defects of a *ΔsreA* mutant are confined to high iron conditions [13,29].

#### Oxidative stress response knockouts

As expected, wild type ROS-detoxifying enzyme activity was lowest under the low iron, low superoxide condition. The SDM of Cat1/2 was less than that of SOD2/3 and the thioredoxin pathway (abbreviated to ThP in Table 3) whenever iron was low. Under low superoxide conditions the model predicted a Yap1 = 0 or SOD2/3 = 0 knockout, but not a Cat1/2 = 0 or Thp = 0 knockout, to be fatal. This observation demonstrates that the model accounts for the redundancy that both catalases and peroxiredoxins reduce H _2_*O*_2_. Under high superoxide conditions, model knockouts of any of the four oxidative stress-related species had a high cell death phenotype due to overwhelming ROS.

The observation that Yap1 or SOD2/3 deletion is more severe than deletion of Cat1/2 or blocking of the thioredoxin pathway is consistent with experimental results from Leal et al. which show that *Δyap1* and *Δsod1/2/3* mutant strains are sensitive to neutrophil-mediated oxidative stress, whereas a *Δcat1/2* strain is not [10]. Yet the observation is inconsistent with a result from the same study which showed that blocking the thioredoxin pathway results in a reduction of *in vivo* hyphal growth similar to deletion of either *yap1* or *sod2/3* [10]. An earlier study, which demonstrated that a *Δcat1/2* mutant showed increased sensitivity to H _2_*O*_2_*in vitro* and delayed growth during infection in a rat model of aspergillosis, further conflicts with the Leal et al. study and provides support for the model predicted phenotype of a Cat1/2 = 0 knockout [9]. Overall, the severity of experimental knockout results seem to be exaggerated by some model predictions. In particular, deletion of either *yap1* or *sod2/3* should not result in high cell death in the absence of oxidative stress, yet under low superoxide conditions the model predicted knockouts a high cell death phenotype for Yap1 = 0 and SOD2/3 = 0 knockouts.

Discrepancies between experimental results and model predictions indicate that important species or interactions may be missing from the model. This may reflect that our understanding of *A. fumigatus* oxidative stress response is still not complete. For instance, there may be unidentified redundancy, some of which could be attributed to LaeA-controlled secondary metabolites which inhibit neutrophil production of NOX [73]. Yet knockout experiments of several of these metabolites as well as *laeA* suggest they may play no role in protecting *A. fumigatus* from neutrophil-mediated oxidative stress [10]. Since model-predicted phenotypes of Yap1 and Yap1-regulated species knockouts are most overstated in low superoxide conditions, it is possible that the model lacks some constitutively active or baseline antioxidants which may be useful for neutralizing ROS produced during normal cellular activities but may not necessarily be helpful in combating oxidative stress. As more research is done to characterize new players in the *A. fumigatus* oxidative stress response network, the oxidative stress response module of this model can be improved and new insights may be gained.

### Model suggests combined blocking of iron uptake and oxidative stress response

Of the four conditions in Table 3, the low iron and high superoxide condition most resembles the environment that *A. fumigatus* cells experience inside a mammalian host. Under this condition, model knockouts which impaire siderophore-mediated iron uptake increased *A. fumigatus* sensitivity to oxidative stress. A similar relationship between high-affinity iron uptake and sensitivity to oxidative stress has been observed in yeast [15]. This observation led us to wonder what the model would predict for ROS levels if combined blocking of siderophore-mediated iron uptake and oxidative stress response were simulated under less rigid external conditions.

Several experimental studies have investigated targeting either siderophore-mediated iron uptake or oxidative stress response, but none have investigated any potential therapeutic gain by combined targeting. TAFC and MirB are promising drug targets since they are easily accessible and unique to fungi [30]. Leal et al. demonstrate proof of concept that using lipocalin-1 to sequester TAFC improves the treatment of topical *A. fumigatus* infection [12]. An anti-cancer drug, PX-12, which is known to block the thioredoxin pathway also shows promise as an anti-*A. fumigatus* drug [10].

**Figure 5:**
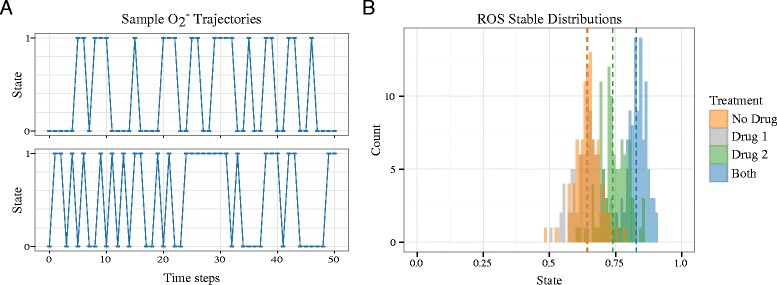
Model results comparing the application of two hypothetical anti-fungal drugs to treat a simulated *A. fumigatus* infection. Iron is fixed at low (0) while superoxide is allowed to randomly toggle between low (0) and high (1). **(A)** Two representative superoxide trajectories. **(B)** ROS stable distributions for no drug, either drug individually, or both drugs together. Vertical dashed lines represent ROS SDMs. Drug 1 targets siderophore-mediated iron uptake. Drug 2 targets oxidative stress response. Simulations are initialized from the state in row 2 of the low iron, high superoxide block in Figure 2.

To investigate the effects of simultaneously inhibiting siderophore-mediated iron uptake and oxidative stress response, we simulated treatment with two hypothetical drugs loosely based on lipocalin-1 and PX-12. Hypothetical drug 1 binds and inactivates TAFC from 50 *%* of fungal cells. Hypothetical drug 2 blocks the thioredoxin pathway in 50 *%* of fungal cells. For these simulations, we held iron fixed at 0 but allowed superoxide to randomly toggle between 0 and 1. This setup recapitulates host defense mechanisms of sustained iron witholding and intermittent respiratory bursts. Figure 5(A) shows two representative superoxide trajectories when random toggling is allowed. Figure 5(B) shows ROS stable distributions from the average of 100 stochastic simulations for treatment with Drug 1, Drug 2, both drugs or neither drug. To simulate the drug treatments with 50 *%* efficacy, we fixed either TAFC, ThP, or both at 0 for 50 of the 100 simulations. Drug 1 alone has no effect, Drug 2 alone increases the ROS SDM from 0.64 to 0.74, and the combination of Drug 1 and Drug 2 further increases the ROS SDM to 0.83. This result suggests that combined targeting of siderophore-mediated iron uptake and the oxidative stress response network may act synergistically to increase fungal cell killing.

## Conclusions

In this study we introduce a stochastic Boolean model of the iron regulatory and oxidative stress response networks in *A. fumigatus*. Model simulations of a population of *A. fumigatus* cells reproduces gene expression patterns in experimental time course data when *A. fumigatus* is switched from a low iron to a high iron environment. In addition, the model is able to accurately represent the phenotypes of many knockout strains under varying iron and superoxide conditions.

We drew three main observations from model analysis. First, the model provides support for the hypothesis that *A. fumigatus* iron regulatory proteins, HapX and SreA, are regulated by iron at the post-translational level. Second, based on discrepancies between model knockout simulations and experimental observations of *A. fumigatus* oxidative stress response related mutants, it is likely that important enzymes or pathways involved in *A. fumigatus* ROS-detoxification remain uncharacterized. And third, impairment of siderophore-mediated iron uptake mechanisms reduces *A. fumigatus* resistance to oxidative stress. This fact could be exploited when designing a treatment strategy.

Deterministic simulation (as well as individual stochastic simulations, data not shown) of the model predicts sustained oscillations under each of the four external conditions (Figure 2). On the other hand when a population of cells is modeled by averaging many stochastic simulations, the model converges to a steady state distribution (Figure 3). Without single cell data, it is unclear how to interpret discrepancies between an individual (deterministic or stochastic) simulation and the average of many stochastic simulations. It is conceivable that, in agreement with individual model simulations, expression of iron homeostasis and oxidative stress response genes in a single *A. fumgatus* cell may continually oscillate, perhaps always overshooting and undershooting ideal intracellular iron levels. A recent study in *E coli* reported damped oscillations in the expression of genes involved in iron homeostasis in a single *E. coli* cell undergoing a switch from high iron to low iron conditions [21]. Future single cell experiments of *A. fumigatus* could shed light on how stochasticity arises in fungal iron regulatory and oxidative stress response networks.

The intended future application of this model is to incorporate it into a multi-scale systems biology model of invasive aspergillosis in the lung. The ultimate goal of the proposed multi-scale model is to capture the effect of the initial inoculum on disease outcome and to allow for the investigation of a variety of therapeutic interventions.

## Methods

### Computational methods

#### Discrete modeling framework

In order to translate the network interactions depicted in the diagram of Figure 1 into a dynamic discrete model, namely a time- and state-discrete dynamical system, the state transitions for each species must be specified by assigning an update rule that describes how the species’s state will be updated at the next time step based upon the states of its inputs at the current time step. Since this is a Boolean model, each species can take on only two states and update rules are Boolean functions. It is convenient to encode update rules in an object called a transition table. As an example, consider the iron-sensing transcription factor SreA. From the literature we know that when intracellular iron levels are low, SreA is kept in an inactive state [59,60]. Based on this we decide the update of SreA should depend on two inputs, its gene *sreA* and the labile iron pool LIP. These interactions are represented in Figure 1 as the two edges incident on SreA. Based on the state descriptions assigned to SreA, *sreA*, and LIP listed in Table 1, we obtain the following table which determines which state SreA will transition to at time step *t*+1 based on the states of *sreA* and LIP at the current time step, *t*.

Using Table 1 to translate the state descriptions into 0’s and 1’s, we obtain the following transition table.

This table encodes the AND function. Both *sreA* AND LIP must be in state 1 at the time *t* for SreA to be in state 1 at time *t*+1. Any Boolean function can be written using only AND, OR, and NOT gates (see Table 2).

For ease of computation, we prefer to work with a mathematical object rather than transition tables or Boolean functions. Any discrete dynamical system can be represented as a system of polynomial equations over a finite field. A model in this form is called a polynomial dynamical system (PDS) and can be analyzed using theory and tools from computational algebra [74,75]. Since each species in a Boolean network model can take on only two states, the finite field for our model is $\mathbb {F}_{2} \cong \mathbb {Z}/2\mathbb {Z}$, i.e., the set of integers {0,1} where addition and multiplication is modulo 2.

The polynomial dynamical system for our model (corresponding to the update rules listed in Table 2) is: $F = (\,f_{1},\ldots,f_{22}): \mathbb {F}_{2}^{22} \to \mathbb {F}_{2}^{22}$ where the variables *x*_*i*_, *i*=1,…,22 are the species and the *f*_*i*_, *i*=1,…,22 are the update functions written as the following polynomials over $\mathbb {F}_{2}$.

$$\begin{array}{ccc} x_{1}= hapX &\quad x_{2}= sreA &\quad x_{3}= \text{HapX} \\ x_{4}= \text{SreA} \;\;&\quad x_{5}= \text{RIA}\;\; &\quad x_{6}= \text{EstB}\;\;\; \\ x_{7}= \text{MirB} &\quad x_{8}= \text{SidA} &\quad x_{9}= \text{TAFC} \\ x_{10}= \text{ICP} \;&\quad x_{11}= \text{LIP} &\quad x_{12}= \text{CccA} \\ x_{13}= \text{FC}^{-Fe} &\quad x_{14}= \text{FC}^{+Fe} &\quad x_{15}= \text{VAC}\;\; \\ x_{16}= \text{ROS} &\quad x_{17}= \text{Yap}1 &\quad x_{18}= \text{SOD}2/3 \\ x_{19}= \text{Cat}1/2 &\quad x_{20}= \text{ThP}\;\; &\quad x_{21}= \text{Fe}^{3+}\\ x_{22}= O_{2}^{-} & & \end{array} $$

$$\begin{aligned} f_{1} & = x_{4}+1 \\ f_{2} & = x_{3}+1 \\ f_{3} & = x_{11} \cdot x_{1}+x_{1} \\ f_{4} & = x_{11} \cdot x_{2} \\ f_{5} & = x_{4}+1 \\ f_{6} & = x_{4}+1 \\ f_{7} & = x_{3} \cdot x_{4}+x_{3} \\ f_{8} & = x_{3} \cdot x_{4}+x_{3} \\ f_{9} & = x_{8} \\ f_{10} & = x_{3} \cdot x_{14} \cdot x_{15}+x_{3} \cdot x_{14}+x_{3} \cdot x_{15}+x_{14} \cdot x_{15} \\ &\quad +x_{14}+x_{15} \\ f_{11} & = x_{21} \cdot x_{5} \cdot x_{9} \cdot x_{7} \cdot x_{6}+x_{9} \cdot x_{7} \cdot x_{6}+x_{21} \cdot x_{5} \\ f_{12} & = x_{3}+1 \\ f_{13} & = x_{8} \\ f_{14} & = x_{11} \cdot x_{13} \\ f_{15} & = x_{11} \cdot x_{12} \\ \end{aligned} $$

$$\begin{aligned} f_{16} & = x_{16} \cdot x_{11} \cdot x_{22} \cdot x_{18} \cdot x_{19} \cdot x_{20} \\ &\quad +x_{16} \cdot x_{11}\cdot x_{22}\cdot x_{18} \cdot x_{19} +x_{16} \cdot x_{11}\cdot x_{22} \cdot x_{18} \cdot x_{20} \\ &\quad +x_{16} \cdot x_{11} \cdot x_{18} \cdot x_{19} \cdot x_{20}+x_{16} \cdot x_{22}\cdot x_{18} \cdot x_{19} \cdot x_{20} \\ &\quad +x_{11}\cdot x_{22} \cdot x_{18} \cdot x_{19}\cdot x_{20}+x_{16} \cdot x_{11}\cdot x_{18} \cdot x_{19} \\ &\quad +x_{16} \cdot x_{22} \cdot x_{18} \cdot x_{19}+x_{16} \cdot x{11} \cdot x_{18} \cdot x_{20} \\ &\quad +x_{16} \cdot x_{22} \cdot x_{18} \cdot x_{20}+x_{16} \cdot x_{18} \cdot x_{19} \cdot x_{20} \\ &\quad +x_{22} \cdot x_{18} \cdot x_{19} \cdot x_{20}+x_{16} \cdot x_{11} \cdot x_{22}+x_{16} \cdot x_{18} \cdot x_{19} \\ &\quad +x_{16} \cdot x_{18} \cdot x_{20}+x_{16} \cdot x_{11}+x_{16} \cdot x_{22}+x_{11} \cdot x_{22} \\ &\quad +x_{16}+x_{11}+x_{22} \\ f_{17} & = x_{16} \\ f_{18} & = x_{17} \\ f_{19} & = x_{3} \cdot x_{17} + x_{17} \\ f_{20} & = x_{17} \\ f_{21} & = x_{21} \\ f_{22} & = x_{22} \end{aligned} $$

#### Incorporating stochasticity

In this study, we use a random update schedule to simulate dynamic behavior. The basic idea behind this approach is to change the deterministic update of each species into a probability of being updated. The stochastic discrete dynamical systems (SDDS) framework was used to generate stochastic simulations [76]. An SDDS is a time- and state-discrete dynamical system which models stochasticity at the functional level by introducing two update probabilities that, together with the update function, specify a probability of transition of a given species at each time step. Let *p*_*i*_*n*^*↑*^ be the probability that species *x*_*i*_ will be updated given that the corresponding update function *f*_*i*_ specifies an increase in state at the next time step. Let $p_{i}^{\downarrow }$ be the probability *x*_*i*_ will be updated given that *f*_*i*_ specifies a decrease in state at the next time step. Then a stochastic discrete dynamical system in *n* variables is a collection of triplets $\left \{f_{i}, p_{i}^{\uparrow }, p_{i}^{\downarrow } \right \}_{i=1}^{n}$ where we may represent the update functions *f*_*i*_ as polynomials over a finite field. Thus a SDDS can be represented as a PDS along with propensity parameters.

The probabilities $p_{i}^{\uparrow }, p_{i}^{\downarrow } \in [0,1]$ for all *i*∈{1,…,*n*} are called the activation propensity and degradation propensity, respectively, of the *i*-th species. If $p_{i}^{\uparrow }=p_{i}^{\downarrow }=1$ for all *i*=1,…,*n* then all species are updated simultaneously at every time step, and the simulation is deterministic. To implement a random update schedule, we let $p_{i}^{\uparrow }=p_{i}^{\downarrow }=0.5$ for all *i*=1,..,*n*, meaning that at each time step each species has an equal probability of either being updated or remaining in its current state. Hence at each time step, some species are randomly selected to be updated whereas others are not. Updating of selected species is done simultaneously at each time step. A “time step” in this model refers to a single round of updates in which the state of any given species can be updated only once. The unit of time step is arbitrary, yet based on comparisons with experimental time course data (Figure 4), we determined each time step of our model corresponds to about 6 minutes of real time.

Analysis of Dynamic Algebraic Models (ADAM), a free web-based software tool which analyzes the dynamics of discrete models using Gröbner bases calculations, was used to generate the above PDS from transition tables and to simulate dynamic behavior using the SDDS framework [77]. ADAM is available at http://adam.plantsimlab.org/.

### Experimental methods

#### *A. fumigatus* strain and growth conditions

The *A. fumigatus* strain used was wild-type AF293. *A. fumigatus* was cultured on glucose minimal media plus agar plates at 37^∘^C for 7 to 10 days until fully conidiated. Spores were harvested by flooding the culture plates with endotoxin-free phosphate-buffered saline solution containing 0.05*%* Tween-20 and swabbing with a sterile inoculation loop to obtain spore suspension. The spores were vortexed and concentrations of spores were determined by counting with a hemacytometer.

#### Incubation and harvesting

*A. fumigatus* was grown in a liquid shaker under iron depleted conditions. 25×10^6^*A. fumigatus* condia were added to standard glucose minimal media plus 0.05*%* Tween-20 but without iron in the trace elements to a final volume of a 25 mL for a final concentration of 1 million spores per mL. Flasks were incubated at 37^∘^C and 200 rpm for 72 hours. Glass flasks were rinsed prior to inoculation with a 0.1 M HCL solution followed by a rinse with double distilled water to remove residual traces of iron. After 72 hours, *A. fumigatus* was shifted from iron depleted to iron replete conditions by adding FeSO _4_ to a final concentration of 10 *μ*M FeSO _4_. *A. fumigatus* was then incubated for another 9 hours.

Mycelia were harvested from triplicate samples at 0 (control), 30, 60, 90, 120, 150, 180, 210, 240, 270, 300, 330, and 360 minutes after the addition of iron. Mycelia were filtered through gauze and immediately flash frozen in liquid nitrogen and stored at −80^∘^C. Frozen mycelia were subsequently ground to a fine powder using a mortar and pestle in the presence of liquid nitrogen.

#### RNA extraction and cDNA synthesis

Total RNA was isolated using a Qiagen RNeasy plant mini-kit. “Protocol: Purification of Total RNA from Plant Cells and Tissues and Filamentous Fungi” was used along with optional on-column DNase digestion step. Extracted RNA was stored at −80^∘^C. RNA integrity was assessed by gel electrophoresis. Concentrations of RNA in each sample were determined by spectrophotometry on a NANODROP 1000 Spectrophotometer. Next, cDNA was synthesized following manufacturer’s instructions (Tetro cDNA Synthesis Kit, Bioline). All incubations were carried out in a thermacycler. Following synthesis, cDNA was stored at −20^∘^C.

#### qRT-PCR

Real time reverse transcription polymerase chain reaction (qRT-PCR) was performed using the cDNA as a template. The constitutively expressed gene *β*-tubulin of *A. fumigatus* was used as the house-keeping gene. See Table 4 for a list of primers for target genes. Real time qRT-PCR was carried out in 20 *μ*L reaction volumes on a BIO-RAD iQ ^*TM*^5 Multicolor Real-Time PCR Detection System machine. The real time qRT-PCR consisted of the following a 3-step protocol: (95^∘^C denaturation for 10 s, 55^∘^C annealing period for 30 s, 72^∘^C extension for 45 s) × 40 cycles. Cycling involved an initial denaturing/polymerase activation step (95^∘^C for 3 min) and a final melting curve analysis (+ 0.5^∘^C ramping × 81 cycles; 30 second incubation between each cycle). SYBR Green (Bioline) was used as the fluorescent reporter molecule in all reactions. Real time qRT-PCR mixes consisted of 1 *μ*L template cDNA to 19 *μ*L master mix. Relative gene expression (fold change from the addition of iron) was quantified using the Pfaffl method and normalized to *β*-tubulin [78]. Results were collected from biological triplicates, and qRT-PCR for each biological replicate was carried out in technical duplicates. Standard errors were calculated to ensure statistical accuracy.

**Table 4:** **Primers used for real-time qRT-PCR**

**Gene**		**Primer sequence (5’-3’)**	**melting**	**Product**
			**Tm (** ^**∘**^ **C)**	**size(bp)**
*β*-*tubuliln*	FP	CTGCTCTGCCATTTTCCGTG	56.8	119
	RP	CGGTCTGGATGTTGTTGGGA	57.3	
*sidA*	FP	TGACGACTCGCCTTTTGTGAA	57.0	474
	RP	TTGCTCGGGTCCATCTCAAC	57.3	
*sreA*	FP	CTCAGTACGATCGCTTCCCC	57.3	297
	RP	GTCCCACAATTACTGCACGA	55.2	
*ftrA*	FP	GGCATGATCGGAGCGTTCTA	57.1	411
	RP	GGCTTGGTTTCCTCCTCGAT	57.2	
*cccA*	FP	GAGCCAAGAGTGAGGCAGAA	57.0	448
	RP	TGCACACCACCCTTGATACC	57.4	

## Additional file

Additional file 1
**Brandon2015_Aspergillus_iron_superoxide.** The Boolean network model of *Aspergillus fumigatus* iron acquisition and oxidative stress response is provided in SBML qual format.
